# Proteomic signals are not equal clinical phenotypes: redefining evidence standards in arbovirus–SARS-CoV-2 cross-reactivity

**DOI:** 10.3389/fimmu.2026.1849848

**Published:** 2026-05-29

**Authors:** Ibzan Jahzeel Salvador Ibarra, Zoila Mora Guzmán, Anahí Jobeth Borrás Enríquez, Juan Alpuche, Gilberto Castañeda-Hernández, Eduardo Pérez-Campos, María Teresa Hernández-Huerta, Hector Alejandro Cabrera-Fuentes

**Affiliations:** 1 Centro de Investigación Biomédica, Universidad Autónoma de Coahuila, Saltillo, Mexico; 2 Centro de Investigación Facultad de Medicina UNAM-UABJO, Facultad de Medicina y Cirugía, Universidad Autónoma “Benito Juárez” de Oaxaca, Oaxaca, Mexico; 3 SECIHTI, Facultad de Medicina y Cirugía, Universidad Autónoma “Benito Juárez” de Oaxaca, Oaxaca, Mexico; 4 División de Estudios de Posgrado e Investigación, Tecnológico Nacional de Mexico/Instituto Tecnológico de Tuxtla Gutiérrez, Chiapas, Mexico; 5 Departamento de Farmacología, Centro de Investigación y de Estudios Avanzados del Instituto Politécnico Nacional, Mexico City, Mexico; 6 División de Estudios de Posgrado e Investigación, Tecnológico Nacional de Mexico/Instituto Tecnológico de Oaxaca, Oaxaca, Mexico; 7 Hospital General de Zona No.1 Dr. Demetrio Mayoral Pardo, Instituto Mexicano del Seguro Social (IMSS), Oaxaca, Mexico; 8 R&D Group, Vice Presidency for Scientific Research and Innovation, Imam Abdulrahman Bin Faisal University, Dammam, Saudi Arabia; 9 División de Estudios de Posgrado e Investigación, Tecnológico Nacional de Mexico/Instituto Tecnológico de Tijuana, Tijuana, BC, Mexico

**Keywords:** antibody-dependent enhancement, arbovirus cross-reactivity, biomarker validation, dengue, immune imprinting, longitudinal cohort studies, proteomics, SARS-CoV-2

## Abstract

The interaction between SARS-CoV-2 and dengue virus represents a biologically plausible yet clinically unresolved challenge in regions where both pathogens co-circulate. Emerging omics-based studies propose that prior SARS-CoV-2 exposure may shape a distinct dengue phenotype through immune imprinting and antibody-dependent mechanisms. However, whether these molecular signals translate into clinically meaningful outcomes remains unclear. This Perspective argues that current evidence remains preliminary and insufficient for clinically actionable interpretation, particularly regarding the conflation of group-level proteomic signals with patient-level clinical phenotypes. Using a recent pilot proteomic study as an illustrative example, we highlight key methodological constraints, including pooled sampling, limited sample size, inadequate control of confounding, and absence of longitudinal and clinical validation. We propose a framework for advancing from exploratory omics observations to clinically interpretable evidence, emphasizing patient-level resolution, temporal dynamics, statistical rigor, functional validation, and integration with standardized clinical endpoints. We also examine the clinical and public health consequences of premature inference, including the potential for premature clinical interpretation, overestimation of disease associations, and challenges in translational interpretation. We conclude that proteomic signals should be regarded as hypothesis-generating rather than predictive until supported by robust, reproducible, and clinically anchored evidence.

## Graphical abstract

**Figure:**
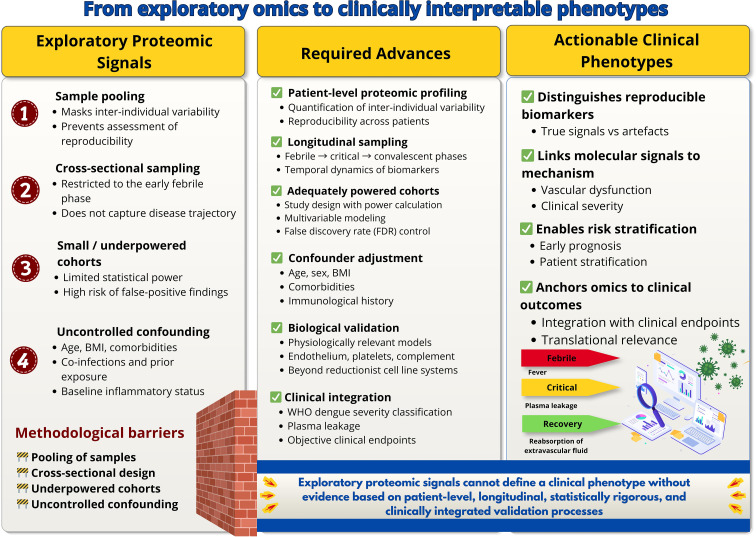
Proteomic signals should not be directly equated with clinical phenotypes without validation: bridging the gap from exploratory omics to clinically actionable insight. This graphical summary illustrates the critical gap between exploratory proteomic signals and clinically interpretable phenotypes in arbovirus–SARS-CoV-2 cross-reactivity studies. Current pilot studies rely on pooled samples, cross-sectional designs, small cohorts, and insufficient control of confounding, limiting reproducibility and clinical inference. To achieve translational relevance, advances are required, including patient-level proteomic profiling, longitudinal sampling across disease phases, adequately powered study designs, rigorous statistical control, biological validation in physiologically relevant systems, and integration with standardized clinical endpoints. Only through these methodological improvements can molecular signals be distinguished from artefacts, linked to mechanisms of disease, and translated into clinically meaningful biomarkers and prognostic tools.

## Introduction

The potential for prior severe acute respiratory syndrome coronavirus (SARS-CoV) exposure to modulate dengue pathogenesis has emerged as a biologically plausible and clinically relevant hypothesis in co-endemic regions (1). Dengue virus (DENV) and SARS-CoV-2 now co-circulate across large regions of the tropics and subtropics, creating new contexts for sequential viral exposure and immune interaction (2). Whether prior SARS-CoV-2 infection alters the clinical course of subsequent dengue represents an important unresolved question, with implications for patient risk stratification and public health surveillance (3). Interest in this problem has been fueled by pilot studies proposing that prior SARS-CoV-2 exposure may shape a distinct or “hybrid” dengue phenotype through immune dysregulation (4), antibody-dependent enhancement (ADE), or persistent inflammatory imprinting (5, 6). Among them, the study by Cruz-Altamirano et al. (4) raises a controversial hypothesis: that prior SARS-CoV-2 infection could modify the host response to dengue in a clinically significant way. This hypothesis is biologically plausible and warrants thorough investigation. However, the main challenge lies not in its plausibility, but in whether the currently available evidence is sufficiently robust to support clinically actionable interpretations. The reliance on a small, pooled proteomic design masks individual biological variance, rendering the 18 identified differentially expressed proteins preliminary. Crucially, there is an uncertain alignment between *in vitro* antibody-dependent enhancement and actual clinical outcomes. Dual-seropositive patients in the NS1/SARS-DENV_IgG group exhibited significantly milder symptoms, which included 0% abnormal hematocrit and 0% vomiting, compared with 50% thrombocytopenia and 50% vomiting in the dengue-only NS1/DENV_IgG controls. Without individual longitudinal validation, these findings do not yet establish a reproducible prognostic association between COVID-19 imprinting and dengue severity. Furthermore, the descriptive clinical data do not clearly support aggravated disease in dual-seropositive patients. These features do not negate the exploratory value of the study, but they do constrain its interpretive reach (4). The study referred to here is not analyzed as an isolated methodological exception, but rather as an illustrative example of the broader translational challenges commonly encountered in omics-based exploratory infectious disease research. Here, rather than solely disputing the conclusions of a single pilot study, we use this case to address a broader problem in arbovirus–SARS-CoV-2 research: what kind of evidence is required to translate exploratory omics observations into clinically interpretable phenotypes (7–9). This Perspective uses this case as an illustrative example to define the broader translational challenges and to delineate the evidentiary standards required for linking omics-derived signals to clinically meaningful phenotypes. Specifically, we examine the methodological and conceptual limits of current evidence and propose a translational framework to distinguish hypothesis-generating molecular signals from clinically actionable inference (7, 10). We argue that this transition requires, at a minimum, patient-level resolution, longitudinal sampling, statistical rigor, control of confounding, biologically relevant functional validation, and anchoring to standardized clinical outcomes (11–13). Until these criteria are met, claims of clinically meaningful SARS-CoV-2–dengue immune imprinting should be interpreted as provisional rather than predictive.

Beyond methodological considerations, the implications of premature clinical inference are substantial. In dengue-endemic regions where SARS-CoV-2 exposure is widespread, misinterpretation of exploratory omics findings as clinically actionable could distort risk stratification, leading to inappropriate allocation of clinical resources or misclassification of patient severity. At the public health level, unvalidated claims of immune imprinting may influence surveillance strategies, confound epidemiological interpretation of disease severity patterns, and complicate the design of vaccination or prevention policies. Furthermore, the absence of robust evidentiary standards risks diverting research priorities toward mechanistically plausible but clinically unsubstantiated hypotheses, delaying the identification of truly predictive biomarkers and actionable pathways. These considerations underscore that improving methodological rigor is not only a technical requirement but a prerequisite for reliable clinical translation and informed public health decision-making. Finally, this perspective does not rule out the biological plausibility of immune interactions between arboviruses–SARS-CoV-2. Rather, we emphasize the distinction between mechanistic plausibility and clinically validated phenotypes, and we highlight the necessary evidentiary steps for their translational interpretation.

## Proteomic pooling and the limits of inference

A central feature of the study is the use of pooled sera for label-free liquid chromatography–tandem mass spectrometry (LC–MS/MS) analysis, an approach that has been shown to introduce systematic bias in clinical proteomics, including both false-positive and missed biomarker identification, and to distort underlying biological averages (14). Although pooling may reduce technical variability and is sometimes employed to mitigate constraints related to sample availability or high biological variation, it fundamentally shifts the level at which biological inference can be made (15, 16). By collapsing multiple individuals into a single composite sample, this approach eliminates inter-individual variance, thereby precluding estimation of variability, identification of outlier-driven effects, and correlation of molecular signals with clinical features. Empirical work in omics-based classification further demonstrates that pooling can compromise analytical performance: increasing pool size is associated with reduced classification accuracy and higher misclassification rates, reflecting the loss of informative individual-level signal (15, 17). In the context of infectious diseases, such heterogeneity is not incidental but integral to pathogenesis. Dengue severity arises from dynamic host–virus interactions shaped by prior immune exposure, genetic background, and metabolic state; averaging across individuals obscures these critical dimensions of biological variation (18, 19). Consequently, the 18 differentially expressed proteins reported cannot be reliably interpreted as consistent features of a patient population; instead, they represent group-level signals lacking defined variance. Critically, the absence of variance estimates and patient-level reproducibility precludes any assessment of biomarker robustness, a key prerequisite for distinguishing true biological signals from cohort-specific artefacts. In the absence of individual-level data, it remains unclear whether these proteins reflect reproducible biological patterns or artefacts introduced by sample aggregation. This limitation does not negate the dataset’s exploratory value; rather, it delineates its interpretative boundaries. Proteomic signals derived from pooled samples can inform hypothesis generation, but they are not sufficient to define clinical phenotypes.

## Statistical constraints in small-scale omics studies

The study comprises 18 participants, with five individuals per infected group, and does not include a formal power calculation (4). As acknowledged by the authors, the analyses are descriptive; however, this design imposes important constraints on downstream interpretation. This limitation is particularly relevant in omics research, where high-dimensional datasets are prone to unstable associations when the number of measured variables greatly exceeds the number of samples (20–22). Pathway enrichment analyses under such conditions are especially sensitive to stochastic variation and sampling effects (23). Proteomic biomarker discovery studies require explicit justification of cohort size and design decisions, aligned with the intended level of inference (24, 25). Similarly, metabolic pathway enrichment results are highly sensitive to background definition, testing strategy, and multiple reporting standards, making control of false discovery rates and transparent enrichment workflows essential rather than optional (26, 27). These candidate molecular differences should not be confused with evidence for clinically relevant biomarkers, which require verification and validation at later stages, beyond exploratory discovery datasets (28–30). Although the reported enrichment of complement/coagulation pathways and transforming growth factor (TGF)-β signaling is biologically plausible and consistent with established features of dengue immunopathology (18, 31), its reproducibility cannot be assumed without safeguards such as multiple-testing correction, false discovery rate control, and external validation (32). In small datasets, nominal p-values may arise from clustering within a limited subset of proteins rather than reflecting stable and reproducible biological effects (33). Accordingly, these findings are better viewed as provisional molecular leads than as statistically robust evidence of a distinct biological or clinical phenotype.

## Confounding and incomplete clinical phenotyping

Serum proteomic profiles are highly sensitive to a range of biological and clinical confounders, including subclinical metabolic disease, variation in body mass index (BMI), undetected co-infections, medication exposure, prior vaccination or infection history, and baseline inflammatory status (12, 34, 35). These factors can shape inflammatory, metabolic, and cytokine-associated signatures independently of prior SARS-CoV-2 exposure (34, 35). Although the study applied general exclusion criteria, it lacks the depth of clinical characterization required to adequately control for these variables. In particular, the absence of detailed metabolic profiling, structured comorbidity stratification, and standardized dengue risk phenotyping limits the interpretation of the observed molecular patterns (34–36). The lack of comprehensive cytokine assessment, including key Th1/Th2 markers such as interleukin (IL)-4, IL-10, and interferon (IFN)-γ, limits the specificity of the observed immune signatures (36, 37), especially given that these immune programs may reflect baseline immunometabolic status, prior immune history, or phase-dependent inflammatory variation rather than a distinct imprint of prior SARS-CoV-2 exposure (36–38). Although LC-MS/MS is highly valuable for broad proteomic profiling, it lacks the analytical sensitivity required for reliable quantification of many circulating cytokines relevant to Th1/Th2 immune characterization (39, 40). Therefore, conclusions about immune polarization based solely on this platform should be interpreted with caution. Highly sensitive multiplex immunoassays, including Luminex-based assays, proximity extension assays such as Olink, and aptamer-based platforms such as SomaScan, can provide more suitable resolution for characterizing immune pathways in translational immunology studies (39, 41–43). Under these conditions, enrichment of Th2-associated pathways cannot be confidently attributed to prior SARS-CoV-2 exposure, as opposed to underlying host variability or unmeasured inflammatory states (12, 35, 38). Because causal interpretation depends on distinguishing exposure-related effects from baseline heterogeneity, specificity must be demonstrated empirically rather than assumed based on biological plausibility alone.

## The central paradox: *in vitro* ADE versus *in vivo* clinical phenotype

The most salient inconsistency in the study lies in the divergence between mechanistic *in vitro* findings and observed clinical outcomes. The proposed “hybrid phenotype” is predicated on the assumption that prior SARS-CoV-2 exposure enhances dengue severity through antibody-dependent enhancement and immune dysregulation (4). However, the descriptive clinical data do not support this interpretation and instead suggest a different, potentially attenuated pattern of early disease expression. In particular, patients in the dengue-only group (NS1/DENV_IgG) exhibited the highest levels of clinical severity, including elevated mean fever, frequent thrombocytopenia, vomiting, and hematocrit abnormalities (44). In contrast, dual-seropositive individuals (NS1/SARS-DENV_IgG) demonstrated a comparatively milder clinical profile, characterized by the absence of hematocrit abnormalities and vomiting, lower rates of thrombocytopenia, and reduced mean fever (45). Although these observations are descriptive and limited by sample size, they do not indicate evidence of exacerbated disease in dual-exposed patients. The clinical variables reported represent only a subset of early manifestations and do not capture the parameters most closely associated with dengue severity. Given that plasma leakage, hemoconcentration, and thrombocytopenia represent central features of dengue severity, their absence in the dual-seropositive group challenges the assertion that prior SARS-CoV-2 exposure potentiates pathogenic outcomes (2). At minimum, the available data indicate that the hypothesis of aggravated disease remains unsubstantiated at the clinical level. This discrepancy is further underscored by the lack of concordance between *in vitro* ADE and patient outcomes. The study demonstrates increased DENV-1 replication in K562 cells in the presence of anti-SARS-CoV-2 sera, consistent with canonical ADE mechanisms (4). *In vitro* ADE systems are valuable mechanistic tools for studying antibody-mediated viral interactions, but their translational interpretation requires integrating them with biologically representative models and validated clinical evidence, since effects observed *in vitro* should not be automatically assumed to predict clinically relevant ADE (46, 47). However, the interpretability of this finding is constrained by the limitations of the experimental system. K562 cells are Fcγ receptor–expressing leukemia cells that, while permissive to dengue infection and capable of supporting viral replication in megakaryocyte-like contexts (48), do not recapitulate the cellular and physiological complexity of dengue pathogenesis, including endothelial barrier dysfunction, platelet consumption, complement activation, and the coordinated immune responses that drive vascular leakage and clinical severity. Recent *in vivo* studies further indicate that antibody-mediated dengue pathogenesis depends on highly specific interactions between IgG and distinct Fcγ receptors, particularly FcγRIIIa on myeloid cells, which drive inflammatory cascades and clinical disease rather than viral replication alone (49). This inconsistency is not unique to the present study. Other reports have also yielded divergent results in both experimental and clinical settings. For example, convalescent SARS-CoV-2 antibodies have been reported to enhance DENV infection *in vitro* and worsen disease in murine models, supporting concerns about cross-reactivity of dengue antibodies (5). Conversely, other studies have shown the opposite pattern: anti-SARS-CoV-2 antibodies inhibit DENV infection, reduce NS1-induced endothelial hyperpermeability, or exhibit dengue-neutralizing activity *in vitro* (50). Similarly, preliminary clinical studies have failed to demonstrate a consistent signal of dengue exacerbation following prior SARS-CoV-2 exposure and, in some cases, have suggested a less severe course (51, 52). Even at the population level, where prior SARS-CoV-2 infection has been associated with a slightly increased risk of subsequent dengue, the relationship between prior exposure, dengue antibodies, and clinically severe dengue remains unresolved (3). These results suggest that cross-reactivity serology alone does not establish a stable or clinically predictive phenotype.

No relationship is established between the magnitude of viral enhancement observed *in vitro* and any clinical parameter of disease severity. Moreover, such assays do not reflect the regulatory networks governing systemic immunity, nor do they account for the compartmentalized nature of host responses. Importantly, no correlation is established between the magnitude of viral enhancement observed *in vitro* and clinical severity in patients. This highlights a fundamental limitation in dengue research: while *in vitro* ADE assays can demonstrate antibody-mediated enhancement of infection, they do not, in isolation, establish pathogenic relevance (53). Translational inference, therefore, requires convergence between mechanistic findings and clinical phenotype across biologically representative systems. Across methodological, statistical, and clinical domains, a consistent pattern emerges: signals generated at the group level are interpreted as patient-level phenomena without the necessary evidentiary bridge.

## The “hybrid phenotype” hypothesis: biological plausibility without clinical validation

The proposed two-hit model, integrating acute dengue vascular pathology with persistent SARS-CoV-2–associated immune dysregulation, is biologically plausible given heterologous immune imprinting and antibody-mediated modulation of viral infections (2, 54). However, the current data do not establish a distinct clinical phenotype, a trajectory toward severe dengue, or a predictive biomarker framework. This limitation is compounded by the absence of longitudinal follow-up. Dengue severity typically manifests during the critical phase (days 4–6), when plasma leakage and hemoconcentration emerge (55), whereas the study captures only the early febrile phase. Consequently, it is not possible to determine whether the observed proteomic signals, including enrichment in the complement and TGF-β pathways, have prognostic relevance. While mechanistic plausibility supports hypothesis generation, clinical phenotypes require reproducibility, temporal validation, and correlation with outcomes; these criteria are not met by the present dataset.

## What would constitute sufficient evidence?

For proteomic observations to support clinically meaningful inferences in dengue following prior SARS-CoV-2 exposure, several criteria must be met. These include molecular profiling at the individual level, with quantification of interindividual variability (25, 56); longitudinal sampling during the febrile, critical, and convalescent phases; adequate statistical power with control of multiple comparisons; systematic adjustment for major clinical and immunological confounders; and validation in biologically relevant functional systems. These molecular findings must be integrated with standardized clinical endpoints (13), such as the World Health Organization (WHO) dengue severity classification (57), objective measures of plasma leakage (58, 59), hematologic dynamics, and prognostic phenotypes (60). In the absence of these elements, proteomic signals can only be considered exploratory and hypothetical, rather than clinically predictive (4, 7, 12).

## Implications for translational biomarker development

In translational medicine, biomarker development requires sequential stages of analytical validity, clinical validity, and clinical utility (61–63). Exploratory omics studies, including genomic, transcriptomic, proteomic, metabolomic, epigenomic, microbiome, and multi-omics approaches, are valuable for identifying candidate biological signals, altered pathways, and mechanistic hypotheses (64–66). However, their findings should not be interpreted as clinical predictors without independent validation, cross-cohort reproducibility, and a demonstrated association with patient-centered outcomes (64, 67, 68). Therefore, it is essential to integrate omics technologies into rigorous translational frameworks to prevent overinterpretation of preliminary molecular signals and facilitate the development of clinically meaningful prognostic tools.

## From exploratory signals to clinical insight

**Figure 1:**
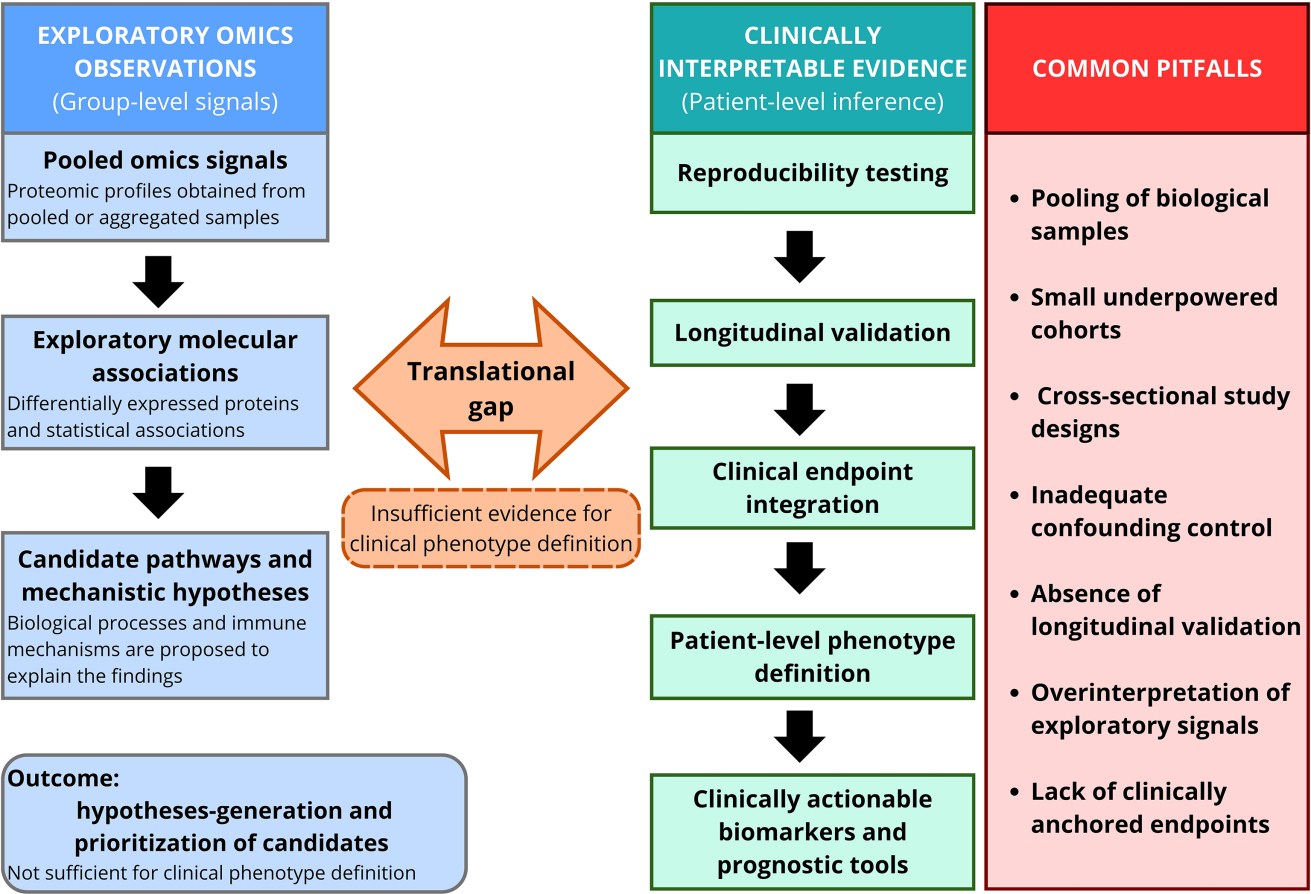
Translational gap between exploratory omics signals and clinically interpretable phenotypes in arbovirus–SARS-CoV-2 studies. Exploratory molecular signals derived from pooled or underpowered datasets may generate biologically plausible hypotheses but are insufficient to define clinically relevant phenotypes without reproducibility testing, longitudinal validation, rigorous control of confounding factors, and integration with standardized clinical outcomes. The image underscores the distinction between group-level molecular observations and actionable, individual-level clinical evidence.

Taken together, the limitations outlined above do not negate the study’s exploratory value but clearly delineate the boundary between hypothesis generation and clinical inference (69). In its current form, the work by Cruz-Altamirano et al. (4) remains insufficiently developed for clinical inference, as the available evidence does not establish reproducible associations between molecular findings and patient outcomes. Bridging this gap requires a shift from reductionist, cross-sectional designs toward integrative, longitudinal approaches capable of addressing inter-individual variability, temporal dynamics, and clinically meaningful endpoints (70). To make these distinctions more explicit, **Table 1** contrasts the defining features of small exploratory omics studies with those of more robust designs that support clinically interpretable inference. **Figure 1** conceptually summarizes the translational gap between exploratory omics observations and clinically interpretable phenotypes in arbovirus–SARS-CoV-2 research, emphasizing the key methodological steps for moving from group-level molecular signals to clinically useful evidence. Advancing the field will therefore depend on study designs that align molecular observations with disease trajectory and severity, incorporate patient-level profiling, use adequately powered cohorts, and perform functional validation within biologically representative systems. Such approaches should be anchored in standardized clinical frameworks, including the WHO dengue severity classification (32, 33), and incorporate mechanistic concepts, such as ADE, that require validation in physiologically relevant systems. A structured framework for this transition, outlining key methodological limitations and corresponding priorities for validation, is summarized in **Table 2**. Without such methodological refinement, there is a risk that early-stage omics observations are prematurely translated into clinical narratives, potentially distorting both research priorities and public health interpretation (73, 74). Collectively, these considerations emphasize that the progression from proteomic signals to clinical phenotypes requires coordinated advances in study design, data integration, and clinical anchoring.

**Table 1:** What distinguishes exploratory omics signals from clinically interpretable evidence in arbovirus–SARS-CoV-2 studies.

Study feature	Pilot exploratory design	More robust comparator design	Added inferential value
Sample structure	Small cohorts, often underpowered and limited in variance estimation (24, 25).	Larger cohorts with explicit cohort-size justification and power considerations.	Improves precision, reduces random effects, and strengthens statistical confidence.
Molecular analysis level	Pooled samples with group-level signals and no inter-individual variance estimation (14, 15).	Individual-level molecular profiling with quantification of inter-individual variability.	Enables assessment of reproducibility, heterogeneity, and biomarker robustness
Temporal design	Cross-sectional sampling during a single disease phase (11, 55).	Longitudinal sampling across febrile, critical, and convalescent stages.	Establishes temporal relationships between molecular changes and disease evolution.
Clinical phenotyping	Limited or descriptive clinical characterization (13, 57–59).	Deep phenotyping with standardized severity criteria, hematologic profiles, plasma leakage, and outcomes.	Supports linking molecular findings to clinically meaningful phenotypes.
Confounding control	General exclusion criteria with limited adjustment for host or clinical factors (12, 34, 35).	Systematic adjustment for demographic factors, comorbidities, co-infections, and immune history.	Improves specificity of observed associations and reduces causal ambiguity.
Statistical framework	Descriptive analyses with limited safeguards against multiple testing (24, 26, 27).	Predefined analytical plan with false discovery rate control, transparent enrichment workflow, and multivariable modeling.	Reduces false positives and improves reproducibility of pathway-level findings.
Biomarker pipeline	Discovery-only dataset (28–30).	Staged discovery, verification, and external validation workflow.	Distinguishes preliminary molecular candidates from clinically credible biomarkers.
Functional validation	Reliance on reductionist *in vitro* systems alone (48, 49).	Validation in biologically relevant systems reflecting Fc receptor biology, vascular dysfunction, complement activation, and platelet responses.	Strengthens mechanistic interpretation and translational relevance.
Clinical interpretation	Suggests plausible molecular differences but limited clinical inference (13, 60).	Integration of omics with standardized clinical endpoints and prognostic outcomes.	Supports stronger inference regarding phenotype, severity, and potential clinical utility.
Overall evidence level	Hypothesis-generating (8, 9, 69).	Better suited for mechanistic understanding and translational inference.	Defines the boundary between exploratory findings and clinically interpretable evidence.

This table contrasts common features of exploratory pilot studies with design elements more consistent with robust biomarker development and clinically interpretable inference. The comparison is intended to clarify not only the limitations of small pooled cross-sectional studies, but also the methodological features required to support stronger mechanistic and translational conclusions.

**Table 2:** From exploratory proteomic signals to clinically actionable insight in arbovirus–SARS-CoV-2 studies.

Domain	Current limitations in pilot studies	Required advancement	Translational implication
Level of inference	Group-level proteomic signals derived from pooled samples without inter-individual variance estimates (14, 15).	Patient-level proteomic profiling with quantification of inter-individual variability and reproducibility across individuals.	Enables distinction between reproducible biomarkers and aggregation artefacts.
Temporal resolution	Cross-sectional sampling restricted to the early febrile phase cannot determine whether molecular signals track disease evolution or predict later severity (18, 36, 55).	Longitudinal sampling across febrile, critical, and convalescent phases.	Establishes temporal linkage between molecular signatures, disease progression, and recovery.
Statistical robustness	Small sample size, absence of power calculation, and limited control of multiple testing (20, 21, 23).	Adequately powered cohorts with multivariate modeling and false discovery rate control.	Reduces type I error and improves reproducibility of pathway-level findings.
Confounding control	Incomplete characterization of metabolic, immunological, and clinical baseline variables (34, 35).	Systematic adjustment for age, sex, BMI, comorbidities, co-infections, vaccination history, and prior flavivirus exposure.	Increases specificity of immune signatures attributable to viral imprinting.
Biological validation	Reliance on reductionist *in vitro* systems (e.g., K562 ADE assays) lacking physiological complexity (48, 49).	Use of biologically relevant models, including endothelial permeability, platelet function, complement activation, and Fc receptor biology.	Links molecular signals to mechanisms of vascular dysfunction and clinical severity.
Clinical integration	Lack of alignment with standardized severity metrics and patient outcomes (71, 72).	Integration omics data with WHO dengue severity classification, plasma leakage, and objective clinical endpoints.	Anchors molecular findings to clinically meaningful phenotypes and prognostic relevance.

Framework for translating proteomic signals into clinically interpretable phenotypes in arbovirus–SARS-CoV-2 studies. Key limitations of current arbovirus–SARS-CoV-2 cross-reactivity studies are contrasted with methodological and analytical advances required for clinical translation. Central to this framework is the distinction between group-level molecular signals and patient-level phenotypes, emphasizing the need for longitudinal design, statistical rigor, control of confounding, and integration with functional assays and clinical endpoints. Representative references supporting key domains are indicated. ADE, antibody-dependent enhancement; WHO, World Health Organization; BMI, Body Mass Index.

## Key translational message

Biological plausibility alone is insufficient to define clinically relevant phenotypes in pathogen-associated cross-reactivity studies, including, but not limited to, studies involving arboviruses–SARS-CoV-2. Translational interpretation requires reproducible evidence at the individual level, longitudinal validation, rigorous control of confounding factors, and integration with clinically significant outcomes.

## Conclusion

The interaction between prior SARS-CoV-2 exposure and subsequent dengue pathogenesis remains an important and relevant question in regions where both viruses co-circulate. Pilot studies, such as that by Cruz-Altamirano et al., provide exploratory observations and point to biologically plausible mechanisms of immune cross-reactivity. However, plausibility should not be confused with clinical validation. Currently available evidence does not establish that prior SARS-CoV-2 exposure modifies dengue severity *in vivo*, nor does it support the definition of a distinct clinical phenotype. This case shows a recurring problem in emerging omics research: the tendency to move from group-level molecular signals to patient-level clinical claims without sufficient supporting evidence. Proteomic observations derived from small, cross-sectional, and pooled datasets can be useful for generating hypotheses, but they should not define clinically relevant phenotypes in the absence of patient-level reproducibility, longitudinal follow-up, and correlation with standardized outcomes. The consequences of conflating exploratory molecular signals with clinical phenotypes extend beyond academic interpretation, with potential impacts on patient management, surveillance systems, and research prioritization in co-endemic settings. The central principle is clear: proteomic signaling is not phenotype. Recognizing this distinction does not diminish the value of exploratory science; on the contrary, it protects its translational integrity. Progress in this field will depend on study designs that integrate individual-level molecular profiling, capture temporal disease trajectories, rigorously control for confounding factors, perform biologically relevant functional validation, and use clinically relevant endpoints. Until such evidence is available, claims regarding SARS-CoV-2 and dengue immune imprinting should be regarded as provisional and hypothesis-generating rather than clinically predictive. Ultimately, translational progress in arbovirus–SARS-CoV-2 research will depend not only on increasingly sophisticated molecular datasets but also on the ability to integrate these signals within reproducible, longitudinal, and clinically anchored frameworks capable of distinguishing biological plausibility from clinically actionable evidence.

## Data Availability

The original contributions presented in the study are included in the article/supplementary material. Further inquiries can be directed to the corresponding authors.
